# Chimeric Cell Therapy Transfers Healthy Donor Mitochondria in Duchenne Muscular Dystrophy

**DOI:** 10.1007/s12015-024-10756-w

**Published:** 2024-07-17

**Authors:** Maria Siemionow, Katarzyna Bocian, Katarzyna T Bozyk, Anna Ziemiecka, Krzysztof Siemionow

**Affiliations:** 1 Chair and Department of Traumatology, Orthopedics and Surgery of the Hand, Poznan University of Medical Sciences, Poznan, 61‑545 Poland; 2 Dystrogen Therapeutics Technology Polska z o.o., Warsaw, 00-777 Poland; 3 Department of Orthopaedics, University of Illinois at Chicago, Chicago, IL 60612 USA; 4 Department of Immunology, Institute of Functional Biology and Ecology, Faculty of Biology, University of Warsaw, Warsaw, 02-096 Poland; 5 Polish Stem Cell Bank, FamiCord Group, Warsaw, 00-867 Poland

**Keywords:** Mitochondria in DMD, Dystrophin expressing chimeric (DEC) cells, Mitochondrial transfer, Mitochondrial fusion, Chimeric mitochondria, DMD therapy

## Abstract

Duchenne muscular dystrophy (DMD) is a severe X-linked disorder characterized by dystrophin gene mutations and mitochondrial dysfunction, leading to progressive muscle weakness and premature death of DMD patients. We developed human Dystrophin Expressing Chimeric (DEC) cells, created by the fusion of myoblasts from normal donors and DMD patients, as a foundation for DT-DEC01 therapy for DMD. Our preclinical studies on *mdx* mouse models of DMD revealed enhanced dystrophin expression and functional improvements in cardiac, respiratory, and skeletal muscles after systemic intraosseous DEC administration. The current study explored the feasibility of mitochondrial transfer and fusion within the created DEC cells, which is crucial for developing new therapeutic strategies for DMD. Following mitochondrial staining with MitoTracker Deep Red and MitoTracker Green dyes, mitochondrial fusion and transfer was assessed by Flow cytometry (FACS) and confocal microscopy. The PEG-mediated fusion of myoblasts from normal healthy donors (MB^N^/MB^N^) and normal and DMD-affected donors (MB^N^/MB^DMD^), confirmed the feasibility of myoblast and mitochondrial fusion and transfer. The colocalization of the mitochondrial dyes MitoTracker Deep Red and MitoTracker Green confirmed the mitochondrial chimeric state and the creation of chimeric mitochondria, as well as the transfer of healthy donor mitochondria within the created DEC cells. These findings are unique and significant, introducing the potential of DT-DEC01 therapy to restore mitochondrial function in DMD patients and in other diseases where mitochondrial dysfunction plays a critical role.

**Graphical Abstract:**

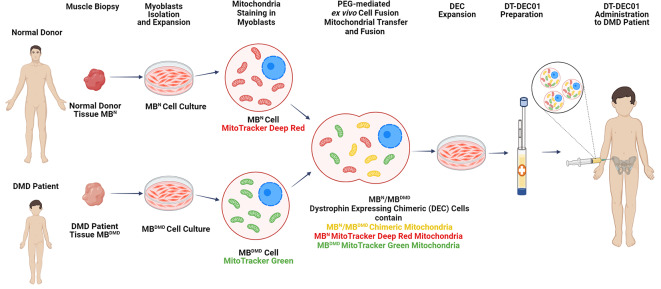

## Introduction

Duchenne muscular dystrophy (DMD) is an X‑linked lethal disease, presenting a significant challenge in medical science due to the mutations in the dystrophin gene, leading to progressive muscle degeneration, weakness and premature death. Despite the standard use of steroids to manage the symptoms, and gene-based therapies aiming at dystrophin delivery, the effective treatments for DMD are currently lacking [1]. Over the years, various cell-based therapies have been explored as potential therapeutic strategies for DMD, aiming to restore dystrophin expression and ameliorate muscle function in the affected individuals [2].

One of the cell-based approaches involved the direct injection of healthy donor muscle stem cells into the muscles of DMD patients [3]. While initially promising, therapies based on the local administration of normal human myoblasts encountered significant obstacles. These included low engraftment efficacy, limited migration, and a high rate of immune rejection, necessitating immunosuppressive regimens to mitigate rejection risks [3–6]. Despite these efforts, only minimal levels of normal dystrophin were detected in the patients’ biopsies following transplantation procedures, highlighting the need for more effective therapeutic strategies [7–11].

Therefore, alternative cell sources, such as satellite cells and mesoangioblasts, have also been investigated for their therapeutic potential in DMD. However, challenges persisted in isolating satellite cells from muscle biopsies, and their integration into muscle tissue remained limited, even after intravenous administration [12]. Similarly, the intra-arterial injection of HLA-matched normal mesoangioblasts failed to demonstrate functional improvement in clinical trials [13].

Recent advancements in stem cell technology have opened new avenues for DMD therapies. Induced pluripotent stem cells (iPSCs) hold the potential promise due to their ability to differentiate into various cell types, including muscle cells [14]. However, the genetic correction of iPSCs, particularly using techniques such as the CRISPR/Cas9 system, presents significant challenges in ensuring the long-term safety and efficacy of the reprogramming process [15].

In light of these challenges, there is a pressing need for innovative approaches to overcome the limitations of existing cell-based therapies for DMD.

Therefore, we propose a novel strategy aimed at addressing these unmet needs through the application of a myoblast-based cell therapy of Dystrophin Expressing Chimeric (DEC) cells, created by the fusion of myoblasts from a normal donor and a DMD patient. This approach offers a unique and universal therapy that overcomes the limitations associated with previously tested cellular therapies for DMD.

We have previously reported significant increase in dystrophin expression in cardiac, respiratory and skeletal muscles in *mdx* mouse models of DMD, which correlated with improved function following systemic intraosseous administration of DEC [16–23]. Furthermore, in the first-in-human clinical study assessing the safety and efficacy of novel DT-DEC01 therapy, we confirmed safety up to 24 months and efficacy demonstrated by improvements in standard functional tests, strength and fatigue resistance which correlated with improvements in Electromyography (EMG) parameters assessed at 12 months after systemic-intraosseous administration of DT-DEC01 to DMD patients [24–26].

While the primary cause of DMD is mutations in the DMD gene, which encodes dystrophin, a protein that helps maintain the integrity of the cell membrane in muscle cells, research studies have suggested that mitochondrial dysfunction may also play a role in the development and progression of the disease [27–29].

Specifically, the mitochondrial dysfunction observed in DMD manifests through several mechanisms, each exacerbating the disease pathology. Energy deficiency, arising from impaired adenosine triphosphate (ATP) production in muscle cells, intensifies muscle weakness and fatigue, whereas mitochondrial dysfunction leads to the overproduction of reactive oxygen species (ROS) and oxidative damage further causing muscle degeneration [30–33]. Moreover, mitochondrial dysfunction triggers inflammatory responses in muscle cells, contributing to the chronic inflammation observed in DMD, which further contributes to muscle weakness and degeneration [34, 35].

Therefore, it is clear that understanding the complex role of mitochondria in DMD pathology is crucial for the development of effective therapeutic strategies. Specifically, targeting mitochondrial dysfunction presents a promising avenue for alleviating muscle weakness and slowing disease progression in DMD. Various pathways enable mitochondrial transfer, including tunnel nanotubes (TNTs), extracellular vesicles (EVs), mitochondrial extrusion, cell fusion, and gap junction channels [30].

To address the need for development of novel therapeutic approaches targeting mitochondrial dysfunction in DMD, our study aimed to further characterize Dystrophin Expressing Chimeric (DEC) cells created by the PEG-mediated ex-vivo fusion. Therefore, we have particularly focused on the creation of chimeric mitochondria and the transfer of normal healthy donor mitochondria to DMD-affected muscles, as a potential therapeutic approach for mitigating DMD symptoms. This research holds promise for advancing our understanding of mitochondrial role in DMD and introduces DEC as a novel therapeutic approach for alleviating disease severity.

## Materials and methods

### Myoblast Isolation

**Fig. 1:**
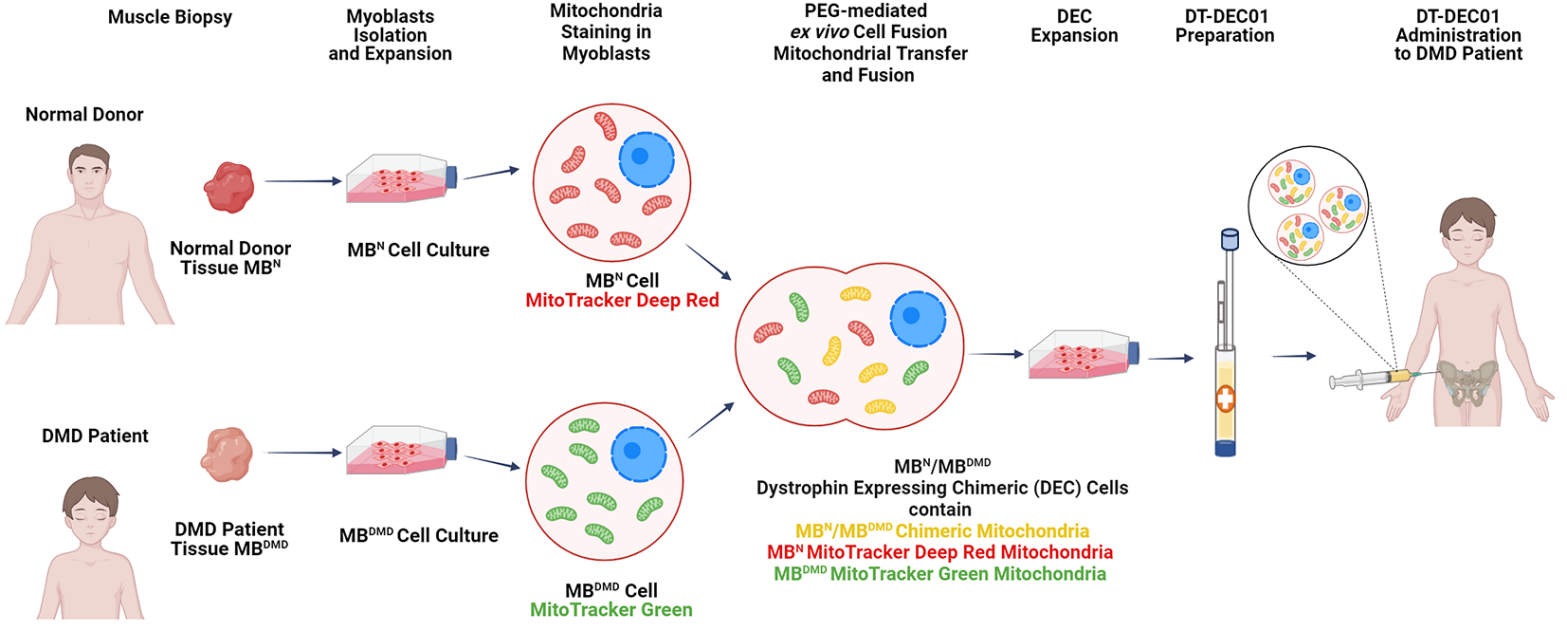
Mitochondrial Transfer and Creation of Chimeric Mitochondria via PEG-mediated Fusion of Human Myoblast Derived from Normal and DMD-affected Donors for Enhancement of Therapeutic Effect of Dystrophin Expressing Chimeric (DEC) Cells. Human myoblasts isolated from tissue biopsies obtained from a normal donor (MB^N^) and a DMD patient (MB^DMD^) were propagated in in vitro cell culture. To assess the fate of mitochondria after cell fusion, normal donor (MB^N^) cells were stained with the mitochondrial dye MitoTracker Deep Red and DMD patient (MB^DMD^) cells with MitoTracker Green. The PEG-mediated fusion resulted in transfer of normal, healthy donor mitochondria and the formation of MB^N^/MB^DMD^ chimeric mitochondria within the Dystrophin Expressing Chimeric (DEC) cells, confirming the enhanced therapeutic potential for DT-DEC01 therapy. The DEC cells were propagated and the DT-DEC01 product was prepared for the systemic intraosseous administration to DMD patient

Human tissue samples were obtained from muscle biopsies of normal donors and DMD patients after signing informed consent according to the protocol based on the approval of the Bioethics Committee at the Regional Medical Council in Poznan, Poland (approval no. 46/2019) and Poznan University of Medical Sciences, Poland (approval no. 672/18). Human myoblast isolation was performed according to well-established protocol as previously described in detail in our publications and literature reports [36–38]. Briefly, skeletal muscle tissue obtained from the tissue samples was minced and subjected to enzymatic digestion using 0.454 U/mL collagenase (Nordmark Pharma GmbH) for 45 min in 33 °C with agitation. The digested tissue was then filtered through a 70 μm mesh to remove debris and centrifuged to obtain a cell pellet. The resulting cell pellet was suspended in culture medium consisting of DMEM (HyClone) supplemented with 1% L-Alanyl-L-Glutamine (Biological Industries), 20% FBS (Lonza) / 10% Human Platelet Lysate ELAREM (PL-Bioscence), 0.5-1% Anti-Anti (Gibco-ThermoFisher), and 12 ng/mL hBFGF (Biotechne). The outline of experimental design of mitochondrial transfer and creation of chimeric mitochondria via PEG-mediated cell fusion is presented on Fig. 1.

### Myoblast Propagation

Following isolation of the tissues obtained from both normal donors and DMD patients, the myoblasts were propagated to achieve a sufficient cell number for subsequent fusion experiments. Myoblasts were cultured in the media supplemented with 6 ng/mL hBFGF (Biotechne). Upon reaching 60–70% confluence, myoblasts were passaged using TrypLE TE Select (Gibco-ThermoFisher). Human myoblasts intended for fusion experiments were collected between passages 5 and 7.

### Detection of Mitochondria in Normal and DMD Donor Cells before Fusion and in the Created DEC Cells

Myoblasts were initially quantified using Trypan Blue (Gibco-ThermoFisher) staining to assess cell viability. Subsequently, the cells were washed in serum-free DMEM media supplemented with 1% antibiotics. Single-staining of myoblasts was performed using fluorescent mitochondrial dyes from Sigma-Aldrich, including 50nM MitoTracker Deep Red (MTDeepRed) and 40nM MitoTracker Green (MTGreen) (Fig. 1). Staining was performed according to the manufacturer’s instructions. Single-stained myoblasts in a 1:1 ratio (MIX) were then fused using polyethylene glycol (PEG 4000, Merck) with dimethyl sulfoxide (DMSO, WAK-Chemie Medical GmbH) in DMEM as previously reported [16].

### Assessment of Cell Fusion by Flow Cytometry

Cell samples from normal and DMD donors were analyzed before and after staining and after the fusion using the FACSLyric flow cytometer and BD FACSuite TM software (BD Biosciences). The analysis was performed by selective gating, and the assessments were based on the analysis of a minimum of 10,000 cells, presented as the percentage of positively stained cells. Excitation and emission wavelengths for MTDeepRed were set at 633 nm and > 650 nm, and for MTGreen were set at 488 nm and 505–570 nm respectively.

### Confocal Microscopy

The cell suspensions (5 × 10^4^/100µl) were applied to polylysine-coated slides and incubated for 30–40 min at 37 °C and 5% CO_2_ to allow for attachment to the slide. Attached cells were treated with an intracellular fixation and membrane permeabilization buffer set (eBioscience™). Incubation was followed by counterstaining with DAPI in PBS (1:1000, Sigma-Aldrich) and sealing the slides with the Fluorescent Mounting Medium (Dako North America Inc.). Fluorescent images were acquired in the FarRed and Green channels using a Nikon A1R MP microscope (NIS-Elements 4.10 software) with a 100x and 60x water immersion lens and pinhole apertures corresponding to 3 μm confocal slices.

Quantitative co-localization of MTDeepRed and MTGreen fluorescent mitochondrial dyes in the chimeric mitochondria was analyzed by Pearson’s correlation coefficient (r) using the Leica integrated program. Based on the interpretation of the assessed r values, correlation coefficients greater than 0.7 indicate that the variables are highly correlated. Correlation coefficients between 0.5 and 0.7 indicate that the variables are moderately correlated [39, 40].

### Assessment of Cell Fusion by FlowSight Imaging Flow Cytometer

Cell samples were analyzed using the FlowSight^®^ Amnis (Cytek^®^ Biosciences). The FlowSight imaging flow cytometer assessed single-cell images in the brightfield channel (Ch01) and in two fluorescence channels: Ch11 for MTDeepRed and Ch02 for MTGreen. The analysis was performed by selective gating, and the values were based on the analysis of 2,000 cells, presented as images of positively stained cells.

## Results

### Confirmation of Mitochondrial Staining by Flow Cytometry

**Fig. 2:**
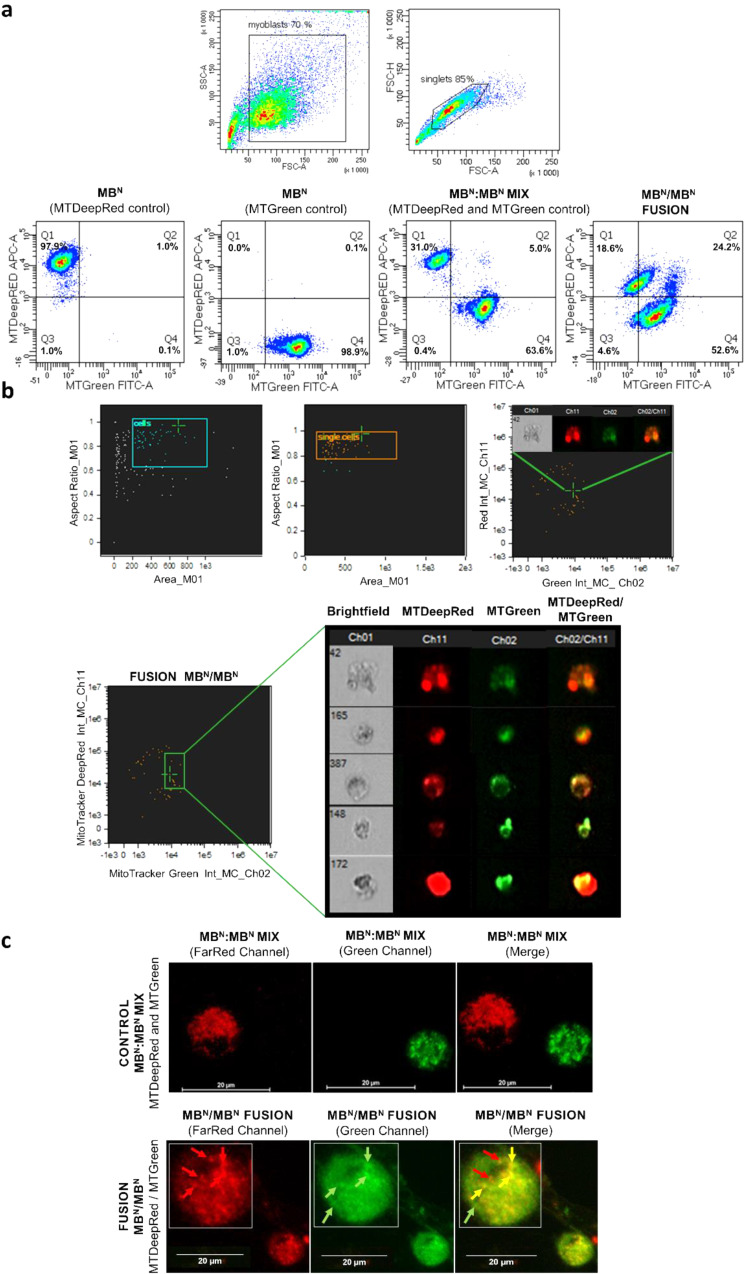
Confirmation of Mitochondrial Transfer and Creation of Chimeric Mitochondria via *ex-vivo* PEG-mediated Fusion of Myoblasts Derived from Normal Human Donors. (**a**) Representative flow cytometry dot plots confirming fusion of the MB^N^/MB^N^ cells stained with the MTDeepRed or MTGreen mitochondrial dyes assessed by FACS. The upper row shows the identification of single myoblasts populations (singlets) among cell debris and aggregates. Lower row shows fluorescence analysis of the single-stained MTDeepRed (Q1) or MTGreen (Q4) mitochondria, the MIX of single-stained MTDeepRed and MTGreen mitochondria (Q1 and Q4) and the overlapping fluorescence of MTDeepRed/MTGreen (Q2) confirming the chimeric state of mitochondria. (**b**) Analysis of the single myoblasts after fusion using the FlowSight^®^ Amnis Imaging flow cytometer. The upper row shows the identification of single cell populations among cell debris and aggregates. The lower row shows (from left to right): the double-positive MTDeepRed/MTGreen signal indicating chimeric mitochondria, the cellular morphology of DEC cells in the brightfield channel (Ch01), the fluorescence image of MTDeepRed (Ch11) and MTGreen (Ch02), and colocalization of the MTDeepRed/MTGreen signal (Ch11/Ch02). (**c**) Representative confocal microscopy images of the myoblasts before and after fusion. The upper row shows the control, MIX of the single-stained MTDeepRed, MTGreen myoblasts and the merged channels (Merge). The lower row shows the fluorescence image of the chimeric myoblast after fusion in the single channels of the FarRed and Green, and the Merge showing overlapping signals of MTDeepRed/MTGreen indicating the chimeric mitochondria. The red arrows denote the mitochondria of normal donor (MB^N^) origin (MTDeepRed), the green arrows indicate the mitochondria of normal donor (MB^N^) origin (MTGreen), and the yellow arrows indicate the chimeric mitochondria of normal donors origin (MB^N^/MB^N^) as shown by the overlapping MTDeepRed/MTGreen fluorescence dyes (magnification 100x/1.49, scale bar 20 μm)

**Fig. 3:**
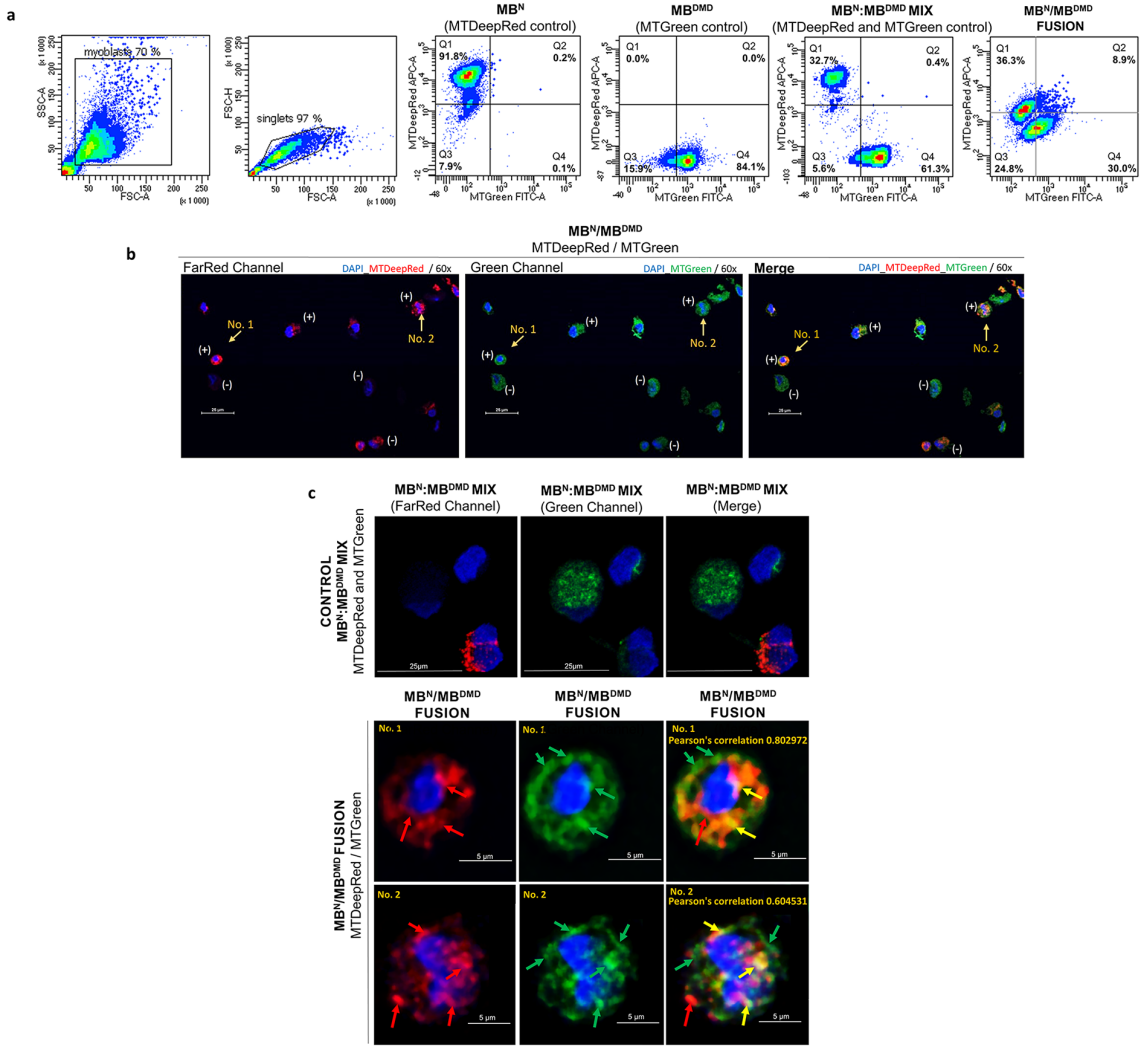
Confirmation of Mitochondrial Transfer and Creation of Chimeric Mitochondria via ex-vivo PEG-mediated Fusion of Myoblasts Derived from Normal and DMD-affected Human Donors. (**a**) Representative flow cytometry dot plots confirming fusion of the MB^N^/MB^DMD^ cells stained with the mitochondrial dyes of MTDeepRed or MTGreen assessed by FACS. The first two dot plots show the identification of single myoblasts populations (singlets) among cell debris and aggregates. Next dot plots show fluorescence analysis of MTDeepRed (Q1) or MTGreen (Q4) mitochondria, the MIX of single-stained MTDeepRed and MTGreen mitochondria (Q1 and Q4) and the overlapping fluorescence of MTDeepRed/MTGreen (Q2) confirming the chimeric state of mitochondria. (**b**) Representative confocal microscopy images of myoblasts after fusion. From left to right, the images show the fluorescence of the stained myoblasts after fusion in a wide field of view in the split channels of the FarRed and Green, and in the merged channels (Merge). Objects marked with (-) are the single-stained normal donor myoblasts (MB^N^) with the MTDeepRed mitochondrial dye or the single-stained DMD patient myoblasts (MB^DMD^) with the MTGreen mitochondrial dye. Objects marked with (+) represent the double-positive chimeric myoblasts (MB^N^/MB^DMD^) with the overlapping MTDeepRed/MTGreen mitochondrial dyes. Yellow arrows indicate the chimeric cells (No. 1 and No. 2), selected for the mitochondrial chimerism analysis shown in the panel below. Nuclei were counterstained with DAPI (blue) (magnification 60x/1.4, scale bar 25 μm). (**c**) Representative confocal microscopy images of the myoblasts before and after fusion. The upper row shows the images of the fluorescence control: the MIX of the single-stained normal donor myoblasts (MB^N^) with the MTDeepRed mitochondrial dye and the single-stained DMD patient myoblasts (MB^DMD^) with the MTGreen mitochondrial dye in the split channels of the FarRed, the Green and the Merge, confirming the absence of fusion by the lack of fluorescence dyes overlap. The rows below represent confocal microscopy images after fusion (MB^N^/MB^DMD^) and show images of the chimeric myoblasts: No. 1 and No. 2 (the second and the third row, respectively). The cells were captured in the single channels of the FarRed where the red arrows denote the mitochondria of normal donor (MB^N^) origin (MTDeepRed) and in the Green channel where the green arrows indicate mitochondria of DMD patient (MB^DMD^) origin (MTGreen). After the merge of the MTDeepRed/MTGreen fluorescence dyes, the chimeric mitochondria are indicated by the yellow arrows. The images on the right, include the Pearson correlation coefficient values for the co-localization of two mitochondrial dyes, confirming the presence of the chimeric mitochondria as shown by the overlapping MTDeepRed/MTGreen fluorescence dyes and determining the degree of mitochondrial chimerism (magnification 60x/1.4, scale bar 5 μm). Nuclei were counterstained with DAPI (blue)

In the realm of investigating mitochondrial transfer subsequent to cell fusion, human myoblasts were stained with mitochondrial dyes to discern chimeric cell formations and the colocalization of post-fusion mitochondria. The initial experiment served as a proof of concept utilizing myoblasts from normal donors (MB^N^/MB^N^). Single staining of 2 × 10^6^ MB^N^ cells each demonstrated 97.9% efficacy with MTDeepRed and 98.9% efficacy with MTGreen staining (Fig. 2a). Subsequently, a second experiment was conducted involving myoblasts from both normal donor and Duchenne muscular dystrophy (DMD) patient (MB^N^/MB^DMD^), simulating conditions analogous to creation of the DT-DEC01 therapy. Single staining of 2 × 10^6^ MB^N^ cells with MTDeepRed exhibited 91.8% efficacy, while staining of 2 × 10^6^ MB^DMD^ cells with MTGreen exhibited 84.1% efficacy (Fig. 3a).

### Confirmation of Myoblast Fusion by Flow Cytometry

Following staining procedures, the standard PEG-mediated fusions were performed. In the first experiment (MB^N^/MB^N^), the fusion efficacy reached 24.2%, and the created chimeric cells exhibited mitochondria of normal donor origin stained either with the MTDeepRed dye or with the MTGreen dye, indicating mitochondrial transfer. Furthermore, colocalization of the mitochondria stained with both MTDeepRed and MTGreen dyes, indicated the chimeric state and chimeric mitochondria formation (Fig. 2a). In the second experiment (MB^N^/MB^DMD^), fusion efficacy reached 8.9% and the created DEC cells displayed either the mitochondria of normal donor origin stained with the MTDeepRed dye or mitochondria of DMD patient origin stained with the MTGreen dye, indicating mitochondrial transfer. Furthermore, colocalization of the MTDeepRed and MTGreen staining was observed, confirming the chimeric state and the creation of chimeric mitochondria (Fig. 3a).

### Confirmation of Myoblast Fusion and Mitochondrial Transfer by FlowSight Imaging Cytometry

The imaging flow cytometry of single cells confirmed the presence of mitochondrial chimerism and transfer after MB^N^/MB^N^ fusion. The created DEC chimeric cells exhibited mitochondria of normal donor origin, stained either with the MTDeepRed dye and with the MTGreen dye. Visualization of the MB^N^/MB^N^ DEC cells confirmed the presence of MTDeepRed and MTGreen mitochondrial dyes, indicating mitochondrial transfer. Colocalization of MTDeepRed and MTGreen mitochondrial dyes in single-cell FlowSight images confirmed the presence of chimeric mitochondria. Variations in the distribution and overlap of the mitochondrial dyes among chimeric cells highlighted the dynamic nature of mitochondrial transfer and the heterogeneity of chimerism within individual cells (Fig. 2b).

### Confirmation of Intercellular Mitochondrial Transfer and Mitochondrial Chimeric State in DEC Cells Created by ex-vivo PEG-mediated Fusion by Confocal Microscopy

Confocal microscopy analysis confirmed intercellular mitochondrial transfer and the creation of chimeric mitochondria in MB^N^/MB^N^ and MB^N^/MB^DMD^ DEC cells. The assessed images depicted the presence of both MTDeepRed and MTGreen dyes within the chimeric cells, indicating mitochondrial transfer. Variability in the overlap of the mitochondrial dyes, indicates heterogeneous levels of mitochondrial fusion (Figs. 2c and 3b, c). Notably, the overlap of the fluorescence staining of MTDeepRed/MTGreen dyes confirmed the chimeric state of the mitochondria after cell fusion (Fig. 2c). Further assessment of confocal images revealed varying degrees of mitochondrial chimerism within DEC cells, with some exhibiting a high level of the colocalization of mitochondrial dyes (Pearson correlation 0.802972), indicating a high degree of the mitochondrial chimerism, while others displayed a lower level of dye colocalization (Pearson correlation 0.604531), suggesting a moderate degree of mitochondrial chimerism (Fig. 3b, c).

## Discussion

Duchenne muscular dystrophy (DMD) is an X-linked genetic disorder characterized by progressive muscle degeneration, inflammation, and fibrosis, ultimately leading to respiratory and cardiac failure and premature mortality of DMD patients. The absence of dystrophin, a structural protein, results in muscle degeneration weakness and functional disability. While there is currently no cure for DMD, the available treatments focus on management of symptoms and enhancement of the quality of life.

To address these unmet needs, based on our experience with development of stem cell therapies in regenerative medicine and reconstructive transplantation [41, 42], we developed the myoblast-based Dystrophin Expressing Chimeric (DEC) cell therapy for DMD patients [16]. We have previously reported the increased dystrophin expression which correlated with reduced inflammation, fibrosis and improved function of cardiac, respiratory and skeletal muscles at 90 and 180 days after systemic-intraosseous DEC administration [17–20]. We have also confirmed biodistribution of human DEC cells to the DMD-affected target organs of heart, diaphragm and gastrocnemius muscle which correlated with reduced pathology and improved organ function confirmed by the echocardiography, plethysmography and the standard functional tests [21, 23].

Moreover, our recent clinical study confirmed DEC therapy safety by the lack of Adverse Events (AE) and Serious Adverse Events (SAE) up to 24 months. Moreover, efficacy of DT-DEC01 therapy was revealed by improvements in standard functional tests and muscle strength which correlated with increased duration of the Motor Unit Potentials (MUP) [24] assessed at 12 months after systemic intraosseous administration of DT-DEC01 therapy to DMD patients [25, 26].

Currently, the focus of DMD research concentrates around delivery of dystrophin either via exon skipping gene therapies or viral vector supported therapies such as microdystrophin and other approaches [43–45].

However, several researchers reported correlation of mitochondrial dysfunction and different diseases including, neurodegenerative disorders, metabolic disorders, and cancer [46]. Mitochondrial damage can induce myopathy through diverse mechanisms, including mitochondrial DNA (mtDNA) deletion mutations, disruptions in Ca^2+^ signaling or loss of oxidative phosphorylation function. Additionally, abnormalities in mitochondrial dynamics, altered mitochondrial structure and morphology, whereas mitochondrial mutations or dysfunction can contribute to the development of muscular dystrophies [30]. Recent literature reports emphasize the role of mitochondria in the mechanism of DMD [47–51]. Experimental study on the *mdx* mice model, highlights the need for new therapeutic approaches which would address mitochondrial dysfunction [52]. In this study cardiomyocytes isolated from *mdx* mice exhibited elevated levels of calcium ions (Ca^2+^) and ROS in response to mechanical stress. These factors could be linked to the initial damage to the sarcolemma and subsequent mitochondrial dysfunction, which resulted in the loss of functional cardiomyocytes leading to the development of heart failure in the muscular dystrophy [52, 53].

Therefore, current research studies focus on the therapeutic role of mitochondrial transfer for enhancement of muscle regeneration and improvement of function [54]. Among numerous methods of mitochondrial transfer and exchange [55–57] several studies have demonstrated the capacity of cell reprogramming. Partial cell fusion between cardiomyocytes and stem cells allowed for substance and mitochondrial exchange, promoting cardiomyocyte reprogramming [58]. Mitochondrial transfer was also achieved between osteocytes via dendritic networks [59] and through the synaptosomes [60]. Methods such as the Sendai virus envelope-based approach enabled controlled cell fusion, influencing mitochondrial transfer rates [61]. Latest reports revealed that mitochondrial transfer from mesenchymal stromal cells to the endothelial cells via tunneling nanotubes under cellular stress, enhanced endothelial engraftment, presenting a novel strategy for vascular cell therapy in ischemic diseases such as the critical limb ischemia and myocardial infarction [62].

The novel approach of our DT-DEC01 therapy [25] created by the fusion of normal and DMD affected human myoblasts, focuses on the delivery of functional dystrophin [16] combined with the transfer of normal, healthy organelles including mitochondria.

Considering the evidence of significant role of mitochondria in the mechanism of DMD disease, the aim of the current study was to confirm the creation of the donor-recipient chimeric mitochondria following *ex-vivo* PEG-mediated fusion of human myoblasts derived from normal and DMD affected donors. To confirm creation of chimeric mitochondria, our approach included mitochondrial staining of the parent myoblasts before cell fusion and assessment of fusion efficacy and colocalization of mitochondria of normal donor and DMD patient origin within the created DEC cells. Notably, two fusion experiments were conducted, a pilot proof of concept experiment assessing feasibility of fusion of myoblasts of normal donor origin and a subsequent fusion procedure of myoblasts derived from normal donor and a DMD patient, mirroring the approach used for creation of the DT-DEC01 therapy. These studies confirmed the feasibility of myoblast fusion and mitochondrial transfer providing valuable insights into the mitochondrial origin within DEC cells.

The fusion of myoblasts mediated by polyethylene glycol (PEG) holds significant promise for the cell-based therapies aimed at treating muscular dystrophies such as Duchenne muscular dystrophy. In this study we observed differences in the efficacy of the cell and mitochondrial fusion depending on the origin of the cells involved. Specifically, higher fusion rates were observed when using myoblasts from the healthy donors compared to the fusion performed between myoblast of a normal, healthy donor with the myoblast derived from the DMD patient. Based on the literature reports [48] these differences may be explained by the presence of dysfunctional mitochondria in the DMD patient cells, which may affect fusion efficacy. Therefore, fusion of myoblasts of healthy donor origin with the DMD-affected myoblasts enhances fusion efficacy and creation of the chimeric mitochondria.

Our study confirmed that creation of DEC cells by the fusion of normal and DMD-affected myoblasts resulted in the creation of chimeric mitochondria and the transfer of healthy donor mitochondria to the created DEC cells. The transfer of functional mitochondria introduces a new potential avenue for the restoration of mitochondrial function in cells containing dysfunctional organelles. Moreover, our analysis of DEC cells after fusion revealed a diverse range of mitochondrial transfer and chimerism levels, indicating the presence of both donor and recipient mitochondria as well as donor-recipient chimeric mitochondria within the same DEC cell. The variability in mitochondrial transfer and chimerism underscores the stochastic nature of the PEG-induced fusion, where the merging of cells occurs in a random fashion. Therefore, application of the Pearson coefficient, to evaluate confocal images of the fused DEC cells, allowed us to assess the correlation between the level of co-localization of the mitochondrial dyes (MTDeepRed/MTGreen) and the level of mitochondrial chimerism. This also enabled the identification of various forms of chimeric mitochondria within the created DEC cells. Moreover, the high value of the Pearson correlation coefficient indicated mitochondria with high colocalization of both MitoTracker dyes, confirming the presence of the mitochondrial chimeric state. In contrast, the lower value of Pearson correlation coefficient indicated the lower degree of co-localization of both MitoTracker dyes, suggesting that the observed double positive mitochondria may be at different stages of mitochondrial fusion. This interesting phenomenon justifies further investigation, to characterize mitochondrial transfer for clinical applications. It should be emphasized that the degree of mitochondrial chimerism observed in DEC cells may have significant implications for the cell’s fate and function. It is possible that cells with higher levels of chimerism may exhibit enhanced metabolic activity and resilience to cellular stress, due to the presence of a greater proportion of healthy mitochondria. This is supported by recent studies which revealed mitochondria as signaling organelles engaged in the intricate intracellular interactions, particularly under stress, with the capacity to induce beneficial responses that restore the function and the homeostasis within the cells [52, 63]. Based on the literature reports, it is clear that many research studies focus on the role of mitochondrial dysfunction in development and progression of DMD. Therefore, this proof of concept study confirming the feasibility of mitochondrial transfer and mitochondrial chimerism formation is encouraging and justifies further investigation into the refinement and optimization of mitochondrial fusion protocol and the assessment of the therapeutic efficacy of the mitochondrial transfer for clinical applications in DMD patients.

## Conclusions

To the best of our knowledge, this is the first report confirming the presence of the chimeric mitochondria in the myoblasts based DEC cells created by *ex-vivo* PEG-mediated fusion. Our study highlights the intricate interplay between cell fusion and mitochondrial transfer within DEC cells. The acquisition of healthy mitochondria via mitochondrial chimerism emphasizes the potential of mitochondrial transfer as a therapeutic strategy for mitochondrial disorders and other cellular pathologies. The unique characteristics of DEC cells will support the regeneration of DMD-affected muscles not only by delivering full-length dystrophin, but also by transferring healthy organelles such as mitochondria from normal, healthy donors, thus enhancing the therapeutic potential of DEC cells. The dual mechanism of action of DT-DEC01 therapy introduces unique therapeutic options for a wide range of the diseases, including Duchenne muscular dystrophy, and other myopathies and neurodegenerative disorders such as Amyotrophic Lateral Sclerosis (ALS) where mitochondrial dysfunction plays a crucial role. Therefore, future studies are guaranteed to provide a more comprehensive understanding of the mechanisms regulating mitochondrial transfer and chimerism within the DEC cells and will be crucial for identifying this unique phenomenon and the full therapeutic potential of DEC cells.

## Data Availability

All data generated or analyzed during this study are included in the published article.
